# Method Comparison and Clinical Performance of Breast Cancer Tumor Markers on Novel Multiplex Immunoassay and Automatized LOCI Technology Platforms

**DOI:** 10.3390/diagnostics13193101

**Published:** 2023-09-30

**Authors:** Lars Schröder, Michael R. Mallmann, Christian M. Domroese, Natalie Wefers, Ramona Dolscheid-Pommerich, Birgit Stoffel-Wagner, Inga Trulson, Kai Vahldiek, Frank Klawonn, Stefan Holdenrieder

**Affiliations:** 1 Department of Gynecology, Ketteler-Hospital Offenbach, 63071 Offenbach, Germany; schroeder.l@ketteler-krankenhaus.de; 2 Department of Gynecology, University Hospital Bonn, 53127 Bonn, Germany; 3 Department of Obstetrics and Gynecology, Faculty of Medicine and University Hospital Cologne, University of Cologne, 50937 Cologne, Germany; 4 Institute of Clinical Chemistry and Clinical Pharmacology, University Hospital Bonn, 53127 Bonn, Germany; 5 Munich Biomarker Research Center, Institute of Laboratory Medicine, German Heart Center, Technical University Munich, 80636 Munich, Germany; 6 Department of Computer Science, Ostfalia University, 38302 Wolfenbüttel, Germany; k.vahldiek@ostfalia.de; 7 Helmholtz Centre for Infection Research, Biostatistics, 38124 Braunschweig, Germany; 8 Center for the Evaluation of Biomarkers, 81679 Munich, Germany

**Keywords:** breast cancer, tumor marker, method comparison, CEA, CA 15-3, CA 125, CA 19-9, AFP, diagnosis, AUC

## Abstract

Tumor marker determinations are valuable tools for the guidance of breast cancer patients during the course of disease. They are assessed on diverse analytical platforms that may be associated with differences according to the methods applied and the clinical performance. To investigate the method dependency and clinical significance of breast cancer protein tumor markers, CEA, CA 15-3, CA 125, CA 19-9 and AFP were measured in a total of 154 biobanked samples from 77 patients with breast cancer, 10 with DCIS, 31 with benign breast diseases and 36 healthy controls using a Millipore multiplex biomarker panel (MP) and an automized version of the routinely used Vista LOCI technology. The markers were compared between methods and investigated for diagnostic performance. CEA, CA 15-3 and AFP showed good correlations between both platforms with correlation coefficients of R = 0.85, 0.85 and 0.92, respectively, in all samples, but similarly also in the various subgroups. CA 125 and CA 19-9 showed only moderate correlations (R = 0.71 and 0.56, respectively). Absolute values were significantly higher for CEA, CA 15-3, CA 125 and AFP in the Vista LOCI as compared with the MP method and vice versa for CA 19-9. The diagnostic performance for discrimination of breast cancer from healthy controls was similar for both methods with AUCs in ROC curves for CEA (MP 0.81, 95% CI 0.72–0.91; LOCI 0.81; 95% CI 0.72–0.91) and CA-15-3 (MP 0.75, 95% CI 0.65–0.86; LOCI 0.67, 95% CI 0.54–0.79). Similar results were obtained for the comparison of breast cancer with benign breast diseases regarding CEA (AUC MP 0.62, 95% CI 0.51–0.73; LOCI 0.64, 95% CI 0.53–0.74) and CA-15-3 (MP 0.70, 95% CI 0.6–0.81; LOCI 0.66, 95% CI 0.54–0.77). Both platforms show moderate to good method comparability for tumor markers with similar clinical performance. However, absolute levels in individual patients should be interpreted with care.

## 1. Introduction

Blood-based protein biomarkers that are associated with tumor development, progression and metastasis are valuable laboratory tools that support the diagnosis, estimation of prognosis, monitoring of therapy response and early detection of recurrence or progression in diverse cancer diseases [1,2,3]. So-called tumor markers often are glycoproteins on the surface of cancer cells that are proteolytically cleaved and shed into the blood stream or intracellular molecules with structural, enzymatic or hormonal activity that are actively excreted or passively released during tumor cell death. Hence, they are part of the high cellular turnover process observed in many rapidly growing tumors [3,4]. Thus, the quantity of tumor marker found in the blood serum or plasma often correlates with tumor stage and mass or with cellular activity and tumor aggressiveness [3,4].

Due to the molecular heterogeneity of breast cancer and various resulting therapeutic regimes, a multitude of tissue and serum biomarkers have been established for breast cancer management, like the tissue expression of the progesterone receptor (PR), the estrogen receptor (ER), the human epidermal growth factor receptor (HER2) and Ki-67 [5]. In addition, several serum protein tumor markers are associated with and adopted in breast cancer management, especially cancer antigen 15-3 (CA 15-3) and carcinoembryonic antigen (CEA) [6], as well as soluble HER2 [7]. Although not currently emphasized in recent guidelines, serum tumor markers continue to be widely utilized in clinical practice, particularly for prognosis assessment and therapy monitoring [3]. Notably, the examination of individual variations from baseline levels before treatment enables important insights into prognosis, risk stratification and subsequent treatment decisions, even among patients whose tumor marker levels appear to be within the normal range, as recently investigated in more than 11.000 patients with breast cancer for the tumor marker CA 15-3 [8]. In particular, the combination of markers could assist in early detection of progression and monitoring treatment in advanced disease [6] and in adjuvant therapy [9]. This is evaluated in ongoing randomized controlled trials, assessing the ability of monitoring breast cancer patients with serum biomarkers alone, compared to image-based monitoring (SWOG S1703 trial, NCT03723928). Due to their limitations in sensitivity and specificity, in screening settings or primary breast cancer evaluations, serum tumor markers have raised considerable debate. Besides the classical breast cancer markers, cancer antigen 125 (CA 125) [10], as a marker for ovarian cancer, carbohydrate antigen 19-9 (CA 19-9) associated with pancreatic lesions and alpha-fetoprotein (AFP), implicated in the management of hepatocellular carcinoma (HCC) and germ-cell tumors [1,11] have been considered as these markers have demonstrated the ability to detect malignancy or recurrence of disease prior to clinical diagnosis [12].

In comparison to many other plasma proteins, tumor markers are only present in minor concentrations in the ng/mL or even pg/mL range, making a very sensitive and (if possible) automatized analytical procedure necessary to enable the fast, reliable, robust, precise, high-throughput and cost-efficient measurement of the markers [13]. In many central hospital laboratories, this is achieved using enzyme immunoassay technologies that are connected with highly sensitive readouts like chemiluminescence on automatized platforms. Thereby, the sensitivity and specificity of the analysis depend mainly on the quality of the antibodies used in the test systems in terms of specificity and affinity to the antigen, the choice of the antibody binding site and its cross-reactivity with other antigens, as well as on the assay formulation with different buffers and the blocking of disturbing factors like heterophilic antibodies or biotin [14,15,16].

While on these automatized platforms, each immunoassay method is optimized for every marker and novel multiplex assays enable the parallel assessment of 10 to 40 protein markers. They are highly appreciated if the volume of the material is small, like in animal experiments or for marker screening approaches, to see if any marker is useful for a diagnostic question [16,17]. Among others, these multiplex assays were applied in recent early cancer detection studies pioneering the combination of liquid biopsy cfDNA markers and tumor markers for better detection rates even in early cancer stages [18,19]. However, it has to be considered that these multiplex methods mainly are research-use-only (RUO) and not in vitro diagnostic certified (IVD-CE) assays and are therefore not applicable for clinical diagnostics in patient material unless validated extensively as so-called lab-developed tests (LDTs) [16,17].

This is all the more relevant as external quality assessment (EQA) trials regularly show the great method dependency of many tumor markers, similarly to many other protein and hormone markers [20,21,22]. In addition, a lacking correlation or even a shift of marker levels is frequently seen in method comparisons on clinical patient samples [22,23,24]. The observed considerable differences in marker levels may play an enormous role for the diagnostic performance, as well as for the monitoring of individual patients during therapy or in the disease surveillance after primary therapy [3,24]. Therefore, we performed the present study using samples from both cancer and non-cancer patients to test for analytical method comparability in both groups and to evaluate the diagnostic performance of both RUO and CE-IVD-based platforms for distinguishing breast cancer from the relevant control group.

## 2. Materials and Methods

### 2.1. Patients

In total, serum samples of 154 women were analyzed, as already described in an earlier study on the diagnostic performance of the whole 24 multiplex markers panel [25]. Among them were 77 patients mainly diagnosed in early Union for International Cancer Control (UICC) stages (31 in stage I, 25 in stage II, 12 in stage III, and 9 in stage IV), prior to surgical or chemotherapy treatment, in addition to 10 patients with precancerous lesions (ductal carcinoma in situ; DCIS). As control groups, 31 patients with benign breast diseases (e.g., ductal hyperplasia, mastopathy and mastitis) and 36 healthy women were included (Table 1) [25].

The inclusion criteria comprised breast cancer patients with active disease at time of venipuncture for whom biobanked samples and clinical data were available. Patients were excluded if the tumor had been removed surgically or had already been treated by ongoing chemotherapy. Furthermore, patients with earlier-diagnosed secondary tumors were excluded, too.

All patients gave informed consent for blood collection in the Biofluid Biobank of the University Hospital Bonn at the Institute for Clinical Chemistry and Clinical Pharmacology supported by the Centre for Integrated Oncology Cologne-Bonn (CIO). This process, as well as the use of the samples for the planned study, were approved by the Local Ethics Committee of the University Bonn (Nr. 319-12).

The consecutive collection of the blood samples was performed between 2010 and 2012 at the Department for Gynecology and Obstetrics of the University Hospital Bonn prior to any therapy and simultaneously with routine blood samplings. The collected blood samples were transported to the Central Laboratory, where they were centrifuged at 4000 rpm (3300 G) for 10 min. Subsequently, the serum samples were aliquoted into polypropylene vials, labeled with a double-pseudonymized code, and archived at −80 °C in the biobank store. For the present study, samples that fulfilled the inclusion criteria were chosen from the biobank without further selection criteria.

### 2.2. Materials and Methods

For the study of the tumor markers on the RUO-multiplex panel, the MilliplexTM MAP Human Circulating Cancer Biomarker Magnetic Bead Panel 1, 96-well plate assay (EMD Millipore^TM^, Billerica, MA, USA) was used and run on the Bio-Plex^TM^ 200 system (Biorad, Hercules, CA, USA). The principle and procedure are described in detail by Hermann et al. [26]. In brief, the immunoassay reaction was performed on the surface of specifically fluorescent-coded magnetic beads that were functionalized with 24 different analyte-specific antibodies. Patient samples and standards, as well as biotinylated detection antibodies and a streptavidin–phycoerythrin conjugate were added to the wells and incubated with the microspheres, subsequently followed by cytometric detection on the Bio-Plex^TM^ 200 system, as described earlier [26]. For the purpose of this comparison, we focused on 5 cancer markers—carcinoembryonic antigen (CEA), cancer antigen 15-3 (CA 15-3), cancer antigen 125 (CA 125), carbohydrate antigen 19-9 (CA 19-9), and alpha-fetoprotein (AFP), cancer—out of the total 24 markers.

The established routine method for tumor marker analysis at the Central Laboratory of the University Clinics Bonn was based on the LOCI™-technology on a Dimension™ Vista 1500 analyzer (Siemens Healthcare Diagnostics, Eschborn, Germany). The internal and external quality control specifications were performed as requested by the guidelines of the German Federal Medical Society (RiliBÄK) [27]. LOCI™-based tumor marker assays are heterogeneous sandwich chemiluminescent immunoassays, working with chemibeads that contain a chemiluminescent dye and sensibeads with a photosensitizer dye. The sandwiches are generated via biotinylated antibodies and chemibeads. The added sensibeads generate immunocomplexes that are detected using a chemiluminescent reaction.

### 2.3. Statistics

Comparisons of the methods in the overall and subgroups of patients with breast cancer and with benign breast diseases were performed via Spearman rank test and Pearson test after log transformation of the values. The differences in marker levels in both methods are presented graphically by boxplots showing median, interquartile ranges and whiskers for each marker and method. The diagnostic performance of single markers was assessed for the discrimination between breast cancer and benign diseases as well as between breast cancer and healthy controls. Significance testing was performed using *t*-tests or Wilcoxon rank–sum tests when the data did not follow a normal distribution.

The regression lines were calculated with linear, Deming and Passing–Bablok regression. Both Deming and Passing–Bablok regression are typically used to compare two measurement methods in clinical chemistry. The linear regression minimizes the sum of squared residuals. In contrast, Deming assumes that measurement errors are present in both the x-values and the y-values. Passing–Bablok does not minimize the residuals. All possible pairs of (x)-(y) points are determined and slopes are calculated using each pair of points. In addition, the areas under the curve (AUCs) of the receiver operating characteristic (ROC) curves were calculated. All comparisons were performed two-sided and the statistical significance was set at *p* < 0.05. The data analysis was performed using R (version 4.2.2; https://www.R-project.org (accessed on 22 March 2023); free software foundation, Inc., Boston, MA 02110-1335, USA).

## 3. Results

### 3.1. Analytical Comparison of the Multiplex and LOCI Methods

**Figure 1:**
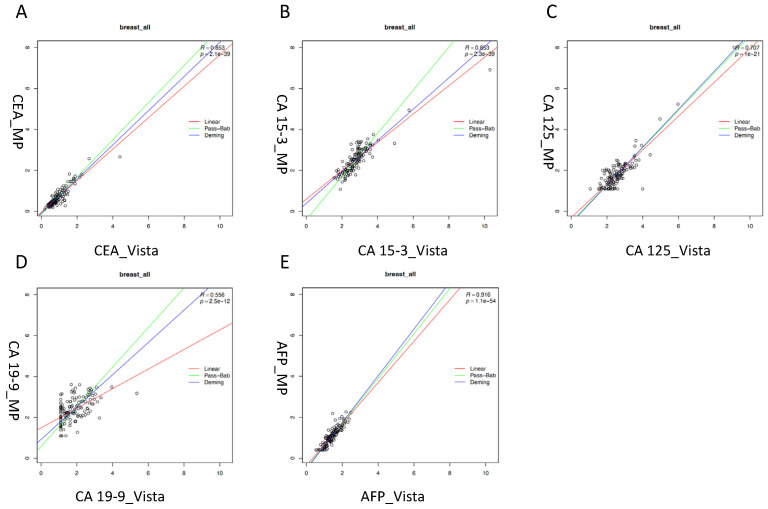
Correlation of tumor markers in all patients measured with Vista LOCI and Millipore multiplex methods: (**A**) CEA (carcinoembryonic antigen). (**B**) CA 15-3 (cancer antigen 15-3). (**C**) CA 125 (cancer antigen 125). (**D**) CA 19-9 (carbohydrate antigen 19-9). (**E**) AFP (alpha-fetoprotein). Linear regression (red line), Passing–Bablok (green line) and Deming (blue line).

CEA, CA 15-3 and AFP showed good correlations between both platforms with correlation coefficients of R = 0.85, 0.85 and 0.92, respectively, in all samples, while CA 125 and CA 19-9 showed only moderate correlations (R = 0.71 and R = 0.56, respectively). For all markers, and particularly for CA 19-9, a considerable scatter of values between both methods was observed. Remarkably, the calculations of the correlations by linear regression, Deming and Passing–Bablok resulted in almost identical curves for CEA, CA 125 and AFP, while there were more variations for CA 15-3 and CA 19-9 (Figure 1).

**Figure 2:**
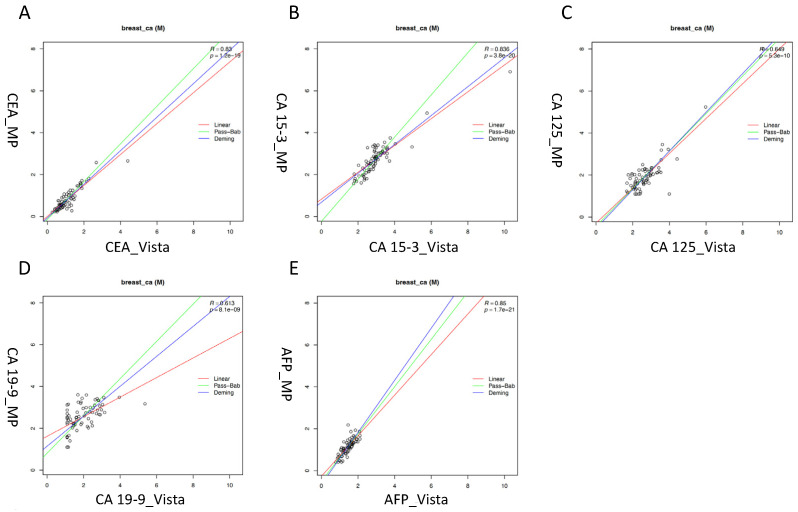
Correlation of tumor markers in patients with breast cancer measured with Vista LOCI and Millipore multiplex methods: (**A**) CEA (carcinoembryonic antigen). (**B**) CA 15-3 (cancer antigen 15-3). (**C**) CA 125 (cancer antigen 125). (**D**) CA 19-9 (carbohydrate antigen 19-9). (**E**) AFP (alpha-fetoprotein). Linear regression (red line), Passing–Bablok (green line) and Deming (blue line).

In the subgroup of patients with breast cancer, similar results were obtained with coefficients of correlation of R = 0.83, R = 0.84 and R = 0.85 for CEA, CA 15-3 and AFP, respectively. Once again, the correlations for CA 125 and CA 19-9 were only moderate (R = 0.65 and 0.61, respectively). Further, considerable scatters of values between both methods were observed for all markers, and particularly for CA 19-9 again (Figure 2).

**Figure 3:**
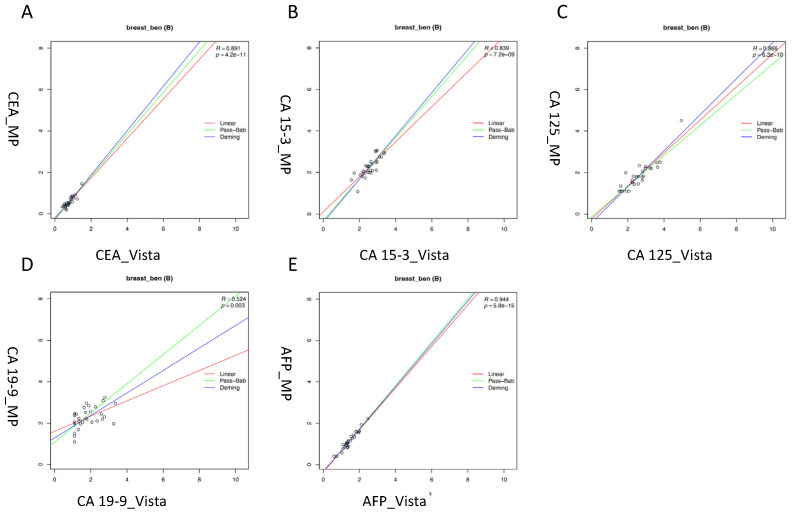
Correlation of tumor markers in patients with benign breast diseases measured with Vista LOCI and Millipore multiplex methods: (**A**) CEA (carcinoembryonic antigen). (**B**) CA 15-3 (cancer antigen 15-3). (**C**) CA 125 (cancer antigen 125). (**D**) CA 19-9 (carbohydrate antigen 19-9). (**E**) AFP (alpha-fetoprotein). Linear regression (red line), Passing–Bablok (green line) and Deming (blue line).

In the subgroup of patients with benign breast diseases, very good correlations were found for CEA (R = 0.89), CA 15-3 (R = 0.84), CA 125 (R = 0.87), and AFP (R = 0.94), respectively, while the correlations were worse for CA 19-9 (R = 0.52). In line with the general findings, a high scatter of values was seen for CA 19-9 (Figure 3).

**Figure 4:**
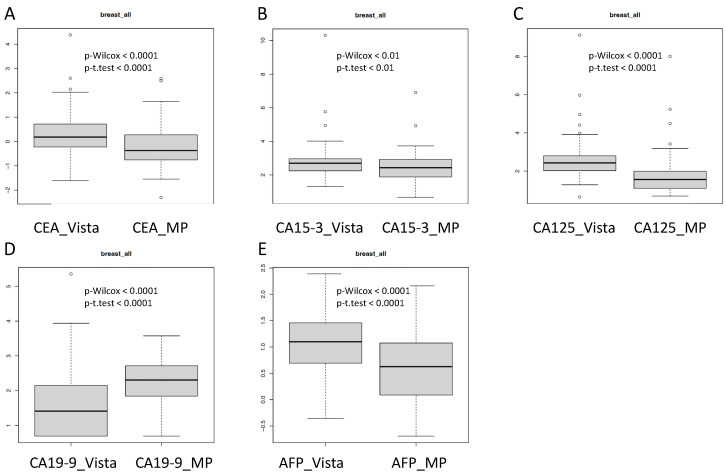
Absolute levels of tumor markers measured with Vista LOCI and Millipore multiplex methods: (**A**) CEA (carcinoembryonic antigen). (**B**) CA 15-3 (cancer antigen 15-3). (**C**) CA 125 (cancer antigen 125). (**D**) CA 19-9 (carbohydrate antigen 19-9). (**E**) AFP (alpha-fetoprotein).

Remarkably, the absolute values of several tumor markers CEA, CA 15-3, CA 125 and AFP were significantly higher on the Vista LOCI platform as compared with the MP method and vice versa for CA 19-9 (Figure 4). This observation shows that the calibration of single tumor markers on the platforms can lead to quite different results and that not all tumor markers are shifted in the same direction by the use of one specific platform.

### 3.2. Comparison of Diagnostic Performance of the Multiplex and LOCI Methods

Diagnostic performance of the tumor markers for the discrimination of patients with breast cancer from healthy controls by use of both methods yielded similar results from the areas under the curve (AUC) of the receiver operating characteristic (ROC) curves. For CEA, the AUCs were 0.81 (95% CI 0.72–0.91) for both MP and LOCI, respectively; for CA 15-3 the AUC was 0.75 (95% CI 0.65–0.86) for MP and 0.67 (95% CI 0.54–0.79) for LOCI. When patients with breast cancer and the relevant control group for differential diagnosis, that is, patients with benign breast diseases were compared, discrimination of the groups was achieved for CEA with AUCs of 0.62 (95% CI 0.51–0.73) for MP and 0.64 (95% CI 0.53–0.74) for LOCI, respectively, and for CA 15-3 with AUCs of 0.70 (95% CI 0.6–0.81) for MP and 0.66 (95% CI 0.54–0.77) for LOCI, respectively (Table 2).

## 4. Discussion

The comparison of five established tumor markers CEA, CA 15-3, CA 125, CA 19-9 and AFP assessed on a novel multiplex immunoassay platform and on an automatized routine method using a LOCI-technology platform in samples from breast cancer patients and control cohorts yields acceptable correlations for CEA, CA 15-3 and AFP while they are lower for CA 125 and CA 19-9. This applies to the comparisons for all patients and for the patient groups with benign breast diseases and breast cancer; for the latter group, CA 125 demonstrated acceptable correlation results, too. This is in line with other method comparison studies investigating the correlation between methods used routinely in patient care [23,24]. However, the good correlations are remarkable as the production quality and lot-to-lot traceability of the tumor marker methods in the multiplex immunoassay panel were expected to be at a lower level due to its classification as a RUO-test. Further, it has to be pointed out that correlations are similar in the cancer and the benign group, and thus can be considered as a general feature independent from the patient cohort. To enable the best comparison between the methods, the values were log-transformed prior to the Spearman rank test and Pearson correlation. For calculation of the regression lines, linear, Deming and Passing–Bablok methods were applied, between which Passing–Bablok was less influenced by outliers and gave the most reliable results.

Nevertheless, a considerable scatter of single values is observed, particularly for CA 19-9, CA 125 and CA 15-3, which are the methods without an international standard available for calibration [28]. This is in line with earlier method comparison studies on these markers using routine laboratory methods [23,24], and studies consistently identifying notable systematic discrepancies across various systems [29,30,31,32,33,34]. Zur et al. [23] conducted a method comparison between the Immulite 200 XPI, Siemens Healthcare and the Dimension Vista 1500 immunoassay (Siemens). Although an acceptable correlation was observed between the methods, notable findings include slope deviations for CA 15-3 and CA 19-9, as well as a wide scatter of values in both low and high concentrations for CA 19-9. Notably, consistently lower concentrations of up to 30% were detected for CA 15-3 on the Dimension Vista platform, whereas for CA 19-9, lower measurements were observed in low concentrations but higher measurements in high concentrations [23]. In contrast, in our investigation, values fitted better for CEA and AFP, for which international WHO standards 73/601 and 72/225 are available [35]. This variability in single samples prevents the introduction of a simple correction factor and illustrates the difficulties for individual value interpretation in case of changes of tumor marker methods. Further, it underlines the necessity to perform double measurement with both methods in case of serial follow up determinations to adjust for the individual level of the patient with the respective method [2,3].

Marlet et al. [29] investigated the analytical performance of the Lumipulse^®^ G1200 (Fujirebio) versus either Kryptor^®^ (Thermo Fisher, Waltham, MA, USA; AFP) or Modular^®^ Elecsys E170 (Roche, Mannheim, Germany; CEA, CYFRA 21-1, CA 125, CA 15-3, CA 19-9, PSA) in 471 serum samples with elevated levels and 100 samples from allegedly healthy subjects. They found a direct transferability for the markers PSA, AFP, CA 125 and CA 15-3; however, a significant bias was observed for CEA and especially CA 19-9, with a mean of difference of 16% and large limits of agreement (−82–113%) in Bland–Altman analysis as well as a slope of 1.52 (95% CI 1.36–1.66) and an intercept of −13.84 in the Passing–Bablok regression analysis for CA 19-9. This is in line with a study [30] comparing six tumor markers on the Lumipulse^®^ G1200 and the AIA^®^ 2000 (Tosoh). Again, CA 19-9 showed a high slope of 1.81 with a Pearson’s correlation coefficient of 0.89. The strong overestimation of the concentration of the Lumipulse^®^ G1200 assay reveals noteworthy bias with method change. The same discrepancies could be observed for CEA in a comparison of four immunoassays in 393 serum samples [36]. Despite an acceptable correlation and no significant bias in Bland–Altman analysis, the relative differences between the assays exceeded 30%.

Importantly, in our investigation, absolute values were found to be significantly higher for CEA, CA 15-3, CA 125 and AFP in the Vista LOCI as compared with the multiplex method and vice versa for CA 19-9. This points at critical issues of calibration for both methods and at difficulties with some markers like CA 19-9 for either of both methods tested. These could be attributable to the complexity of the assays as a whole or the challenging antibody selection and production for the immunoassays.

Similar differences between assays as well as variabilities within laboratories using the same assays and platforms are regularly observed in external quality assessment (EQA) schemes and hint at a necessary better standardization and harmonization of immunoassays for tumor markers [20,21,22,37]. The analyzed EQA data reveal substantial variations in the results of single tumor markers, such as CA 19-9, with a mean coefficient of variation (CV) exceeding 25% [15,20]. The utilization of EQA data can serve as a valuable resource for comprehending the analytical variability of test systems employed in individual laboratories. This understanding is particularly relevant for interpreting and discussion of clinical results from patient care, as well as the consideration of their limitations when obtained from different laboratories. Additionally, it could aid in critically evaluating the applicability of the recently discussed reference change values (RCV) [38,39], which are based on within-subject biological variation and the analytical variation among assumed healthy individuals [40].

Notably, in our study, despite the varying levels observed for individual tumor markers, both methods exhibited comparable diagnostic performance, leading to similar conclusions that could be drawn. Christenson et al. [41] investigated the methodological and clinical concordance of the Dimension Vista^®^ (Siemens Healthcare) and the ADVIA Centaur^®^, Siemens Healthcare (for CA 15-3, CA 19-9, CA 125), as well as the Access^®^, Beckmann Coulter (for CEA) and the Axsym^®^, Abbott assay (for AFP). Despite acceptable agreement between the methods in the Passing–Bablok analysis, the scattering of 10–40% of the values in residual plots showed a mentionable variability. However, based on their data for monitoring patients, when levels of tumor markers are categorized as stable or showing an increase or decrease beyond their RCVs, there was an acceptable clinical correlation observed. However, always keeping in mind that a significant tumor marker change is based on a standard 95% probability and a clinical interpretation using RCV is limited as discussed extensively by Rossum et al. [38], it has to be pointed out that the construction of reference change values (RCVs) is based on the biological variation observed in healthy individuals. However, it is important to note that the situation in patients with cancer differs significantly. In this population, the levels of biomarkers can be influenced by factors such as the underlying disease itself, the administration of adjuvant or palliative therapies, as well as associated toxicities or comorbidities [38].

Nevertheless, this emphasizes the relevance of performing methodical and clinical comparisons in parallel in order to estimate the effect of a method change for the clinical interpretation. Several multicenter studies [24,42,43] investigated the analytical and clinical performance of the UniCel Dxl 800 assay, Beckmann Coulter in reference to the Elecsys 2010 system, Roche. The method comparison for CA 19-9 [42] exhibited strong analytical (R = 0.94) and clinical correlation, as evidenced by a slope of 1.02 and intercept of 0.05 in Passing–Bablok analysis conducted on 1765 clinical samples with minimal scatter observed in low concentrations. However, the spread of values increased at concentrations around 40 kU/L. Both methods demonstrated high diagnostic accuracy for CA 19-9 of pancreatic cancer and comparable AUCs to our results for breast cancer (AUCs of 0.67 and 0.60, respectively), when compared to a control cohort with benign breast disease. Almost identical conclusions were drawn for CA 125 [24]; however, it offered the best discriminative ability for ovarian cancer from the respective benign control cohort. Analytical comparison of the two methods for CA 15-3 [43], however, presented with a weaker correlation (R = 0.81) and a slope of 1.35 and intercept of 0.30, as well as a larger scatter pattern on lower and higher concentrations. The clinical correlation of the methods was good and the diagnostic accuracy for breast cancer against benign control was reported with an AUC of 0.71, almost identical to the AUC (0.7) for the Millipore multiplex method investigated in our study.

However, all studies observed considerable differences for individual patients. Therefore, it is urgently recommended to perform duplicate measurements using both the old and new methods when transitioning instruments or methodologies within a laboratory.

The limitations of the present study comprise the retrospective design, the limited patient number, the comparison of only two methods and the lack of a validation cohort. Thus, further validation is recommended if the RUO method is used for clinically relevant decision making. On the other hand, this study provides some important benefits as the methodical comparison was combined with a clinical comparison with cancer and non-cancer cases being included. Further, standardized blood collection at time of active disease, standardized preanalytical sample handling and high-quality controlled analysis, and the prior preanalytical and analytical validation of the multiplex assays [26], as well as independent statistical evaluation assure a highly informative correlation study. In addition, guidelines for reporting diagnostic accuracy studies (STARD) [44] were followed.

## 5. Conclusions

Both platforms show moderate to good method comparability for tumor markers with similar clinical performance for differential diagnosis of breast cancer. However, absolute levels in individual patients have to be interpreted with caution. Most importantly, methods must be maintained when monitoring the course of disease using serial marker determinations.

## Figures and Tables

**Table 1:** Patient characteristics (adapted from [25]).

		Number	% of Cancers	Age Median	Age Range
**Breast Cancer**	All	77	100	58.7	31.3–85.8
	Stage 1	31	40.3	58.7	36.4–85.4
	Stage 2	25	32.5	62.3	41.3–85.8
	Stage 3	12	15.6	49.5	32.6–74.9
	Stage 4	9	11.7	66.9	31.3–76.4
**DCIS**	Precancerous	10		53.5	39.5–71.0
**Benign breast** **disease**		31		53.8	26.6–85.4
**Healthy controls**		36		42.9	20.1–78.1

**Table 2:** Areas under the curve (AUCs) for breast cancer versus healthy controls and versus benign breast diseases. CEA (carcinoembryonic antigen), CA 15-3 (cancer antigen 15-3), CA 125 (cancer antigen 125), CA 19-9 (carbohydrate antigen 19-9), AFP (alpha-fetoprotein).

	Breast Cancer vs. Healthy		Breast Cancer vs. Benign Disease	
	Vista AUC (95% CI)	MP AUC (95% CI)	Vista AUC (95% CI)	MP AUC (95% CI)
**CEA**	0.81 (0.72–0.91)	0.81 (0.72–0.91)	0.64 (0.53–0.74)	0.62 (0.51–0.73)
**CA 15–3**	0.67 (0.54–0.79)	0.75 (0.65–0.86)	0.66 (0.54–0.77)	0.70 (0.60–0.81)
**CA 125**	0.63 (0.51–0.74)	0.74 (0.62–0.86)	0.54 (0.41–0.67)	0.55 (0.42–0.68)
**CA 19–9**	0.64 (0.51–0.77)	0.75 (0.65–0.86)	0.49 (0.36–0.61)	0.64 (0.53–0.75)
**AFP**	0.74 (0.60–0.87)	0.79 (0.67–0.91)	0.50 (0.37–0.63)	0.51 (0.38–0.63)

## Data Availability

Not applicable.
